# Neurenteric Cyst Presenting with Bleeding Per Rectum

**DOI:** 10.21699/ajcr.v7i4.454

**Published:** 2016-09-01

**Authors:** Taruna Yadav, Padam Parmar, Kamal Nain Rattan

**Affiliations:** 1 Department of Radiodiagnosis,PT BDS PGIMS, Rohtak, Haryana; 2 Department of Pathology,PT BDS PGIMS, Rohtak, Haryana; 3 Department of Pediatric Surgery, PT BDS PGIMS, Rohtak, Haryana

**Keywords:** Duplication cyst, Neurenteric cyst, Malrotation, Bleeding per rectum

## Abstract

Neurenteric cyst in the thoracic cavity may produce a myriad of clinical features. We report a 7-month-old girl who presented with significant bleeding per rectum. On imaging, a mediastinal cystic structure with air-fluid levels was evident with cervico-thoracic vertebral anomalies. The cyst was excised and histopathology showed intestinal mucosal lining with heterotopic pancreatic tissue confirming the diagnosis of neurenteric cyst.

## CASE REPORT

A 7-month-old female baby brought to the pediatric surgery outpatient department with intermittent bleeding per rectum since last 10 days. The baby was diagnosed to have a mediastinal cystic lesion on antenatal scan. Baby was delivered at term without any perinatal problems. During past 7 months, 3 episodes of lower respiratory tract infections occurred which were treated medically.

On examination, child had mild tachypnea along with pallor. Laboratory tests showed anemia (7g/dl) with a normal total leukocyte and platelet count. Chest radiograph revealed multiple cervico-thoracic vertebral segmentation anomalies. Left side upper rib anomalies were also seen along with associated scoliosis with concavity to left side. A well defined radio-opacity was seen projecting over cardiac shadow extending into left hemithorax. Few small bowel loops were seen in right upper quadrant of abdomen (Fig.1). A contrast enhanced computed tomography (CECT) scan revealed a well-defined cystic lesion with mucosal enhancement in posterior mediastinum with air fluid level extending to left side (Fig.2). Esophagus could be traced upto mid thorax only. Stomach could not be clearly delineated among all bowel loops in upper abdomen as small bowel loops were also seen in Morrison’s pouch and retroperitoneum. SMA-SMV relationship could not be relied upon as pancreas was displaced anteriorly by the bowel loops. Azygos continuation of IVC was seen draining into SVC. Hepatic veins were seen to drain into right atrium via a short common channel. Heart and descending thoracic aorta were displaced anteriorly by the cystic lesion along with compression of left lung. Multiple vertebral fusion and segmentation anomalies were seen extending from C5 to T4 vertebrae, along with left upper rib abnormalities and associated scoliosis. No obvious intra-spinal extension of the lesion was seen (Fig.3).

**Figure:**
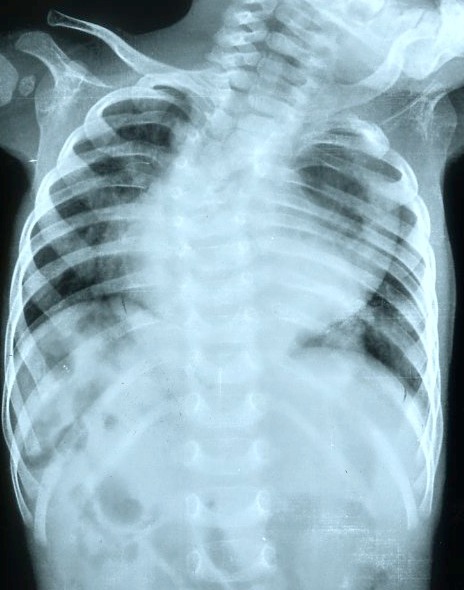
Figure 1: Chest radiograph showing a lesion projecting over cardiac shadow with associated cervico-thoracic scoliosis, multiple anomalies of lower cervical, upper thoracic vertebrae, left upper ribs.

**Figure:**
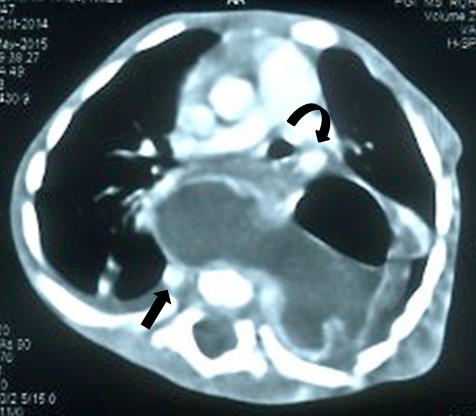
Figure 2: Axial CECT showing a well-defined posterior mediastinal cystic lesion is seen with air fluid levels showing mucosal enhancement. Cystic mass is extending in between and is displacing descending thoracic aorta anteriorly (curved arrow), azygous vein posteriorly (straight arrow).

**Figure:**
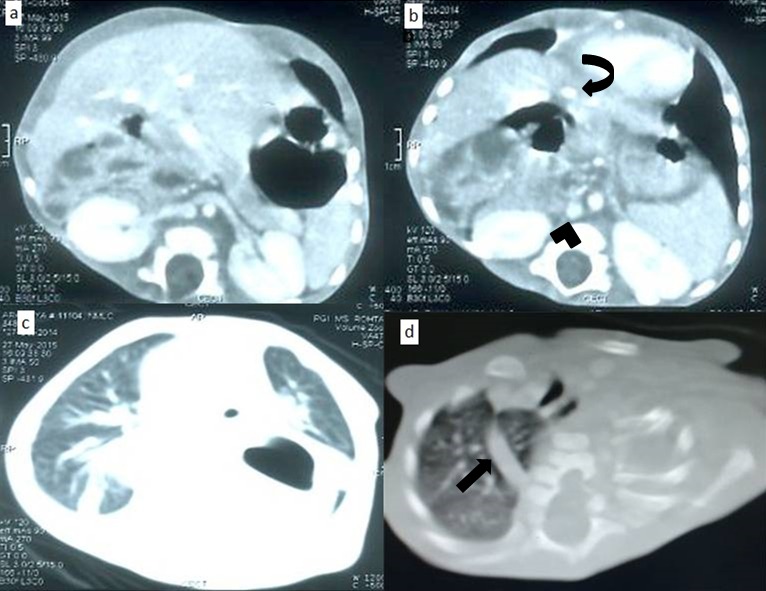
Figure 3: Axial CECT of chest and upper abdomen. (a) Bilateral renal veins are draining into inferior venacava (IVC). Small bowel loops are noted in retroperitoneum in right upper quadrant. (b) All hepatic veins drain into a common channel (curved arrow) draining into right atrium. IVC is seen to continue as azygous vein (arrowhead) (c) Cyst is causing compression of adjacent left lung. (d) CT confirming segmentation, fusion anomalies of lower cervical, upper thoracic vertebrae, left upper rib anomalies. A prominent azygos arch (straight arrow) is evident.

An upper gastrointestinal (GI) contrast study was done which showed normal location of stomach and no opacification of mediastinal lesion by contrast (Fig.4). Meanwhile, child had 3 episodes of malena during the preoperative hospital stay and intermittent hemodynamic instability with drop in hemoglobin which was managed with blood transfusions. After hemodynamic stabilization, left lateral thoracotomy was done. Intraoperatively, a large mediastinal cyst was seen communicating with a proximal jejunal loop via a thin tubular tract. Communication was present on the mesenteric side of jejunal loop. Cyst was resected completely along with a small part of jejunum followed by end to end jejunal anastomosis (Fig.5). Bowel malrotation was present, which was corrected. Histopathological examination of the cyst showed jejunal type of intestinal mucosal lining. Islands of heterotopic pancreatic tissue were seen in the wall of the communicating tract between jejunum and mediastinal cyst (Fig.6).

**Figure:**
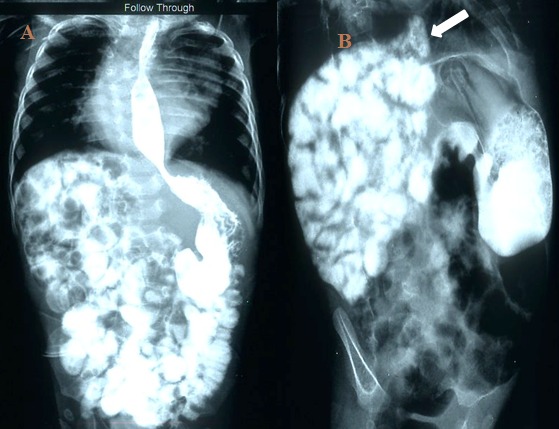
Figure 4: An upper GI series (a) showing normal intrabdominal location of stomach, small bowel loops in right upper quadrant. (b) Postoperatively, retrospective examination of an oblique view from this series showed a contrast outlined tract arising from small bowel loops and extending above the diaphragm (white arrow).

**Figure:**
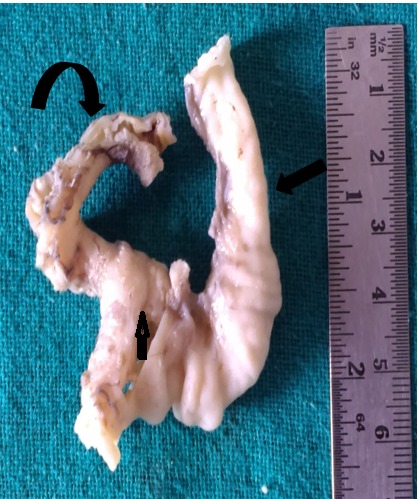
Figure 5: Excised gross specimen showing mediastinal cyst (thick straight arrow), resected part of jejunum (curved arrow) and the communicating tract (thin straight arrow).

**Figure:**
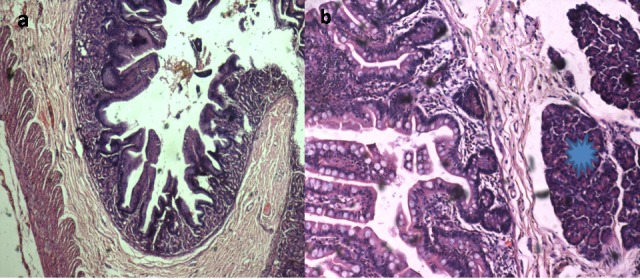
Figure 6: Histopathology sections (a) cyst wall showing intestinal mucosal lining (b) heterotopic pancreatic tissue in the cyst wall (asterisk).

The baby recovered well and was discharged on 10th postoperative day. On a follow up of 6 months after the surgery, the child is thriving well. Postoperatively, on retrospective examination of the upper GI contrast study we observed that an oblique view from this series showed a contrast outlined tract arising from small bowel loops and was extending slightly above the diaphragm (Fig.4).

## DISCUSSION

Mediastinal cysts contribute 15%–20% of all mediastinal masses and include bronchogenic cysts, esophageal duplication cysts, pericardial cysts, neurenteric cysts, meningocele, thymic cysts, cystic teratoma, and lymphangioma.[1,2] Exact characterization of these cystic lesions on imaging may be sometimes difficult and a final diagnosis is made after histopathological examination.

Symptoms may be present at birth if the mediastinal cyst is large in size. Infants may present later with a wide variety of symptoms based on size, location and type. Respiratory distress (dyspnea, stridor, wheezing) can occur due to mass effect. These cysts may cause recurrent chest infections in infants.[7] Our patient did not have any respiratory distress at birth, however had three episodes of lower respiratory tract infections which required treatment with intravenous antibiotics. Hemorrhagic complications like hemoptysis or hematemesis or malena can occur due to heterotopic gastric or pancreatic tissue within the cyst wall causing erosions and peptic ulceration.[1] In our case also, pancreatic tissue was seen in the wall of communicating tract between jejunum and mediastinal cyst resulting in malena episodes. Additionally, spontaneous hemorrhage may occur within the cyst itself.[3,5]

Azygos continuation of the IVC means absence of the hepatic segment of the IVC with azygos continuation. The prevalence is 0.6% .The renal portion of the IVC receives venous return from bilateral kidneys and passes posterior to the diaphragm to enter the thorax as the azygos vein. In our case, all three hepatic veins were draining via a common channel into right atrium. The prominent azygos vein joins the superior vena cava at the normal location.[4]

Primary management of neurenteric cysts is surgical resection with the goal of total resection. Partial resection due to vertebral anomalies or extensive adhesions is the main reason for the recurrence.[5]

In conclusion, neurenteric cysts have a wide spectrum of associated anomalies with different clinical presentation. This case illustrates the importance of multimodality imaging before surgical strategy planning. Clinico-radio-pathological correlation is a must to reach a final diagnosis in such complex cases for better patient management.

## Footnotes

**Source of Support:** Nil

**Conflict of Interest:** None declared

